# Noncatalytic Domains in DNA Glycosylases

**DOI:** 10.3390/ijms23137286

**Published:** 2022-06-30

**Authors:** Natalia A. Torgasheva, Evgeniia A. Diatlova, Inga R. Grin, Anton V. Endutkin, Grigory V. Mechetin, Ivan P. Vokhtantsev, Anna V. Yudkina, Dmitry O. Zharkov

**Affiliations:** 1 SB RAS Institute of Chemical Biology and Fundamental Medicine, 8 Lavrentieva Avenue, 630090 Novosibirsk, Russia; ashatan314@gmail.com (N.A.T.); e.diatlova@g.nsu.ru (E.A.D.); grin@niboch.nsc.ru (I.R.G.); aend@niboch.nsc.ru (A.V.E.); mechetin@niboch.nsc.ru (G.V.M.); i.vokhtantsev@g.nsu.ru (I.P.V.); ayudkina@niboch.nsc.ru (A.V.Y.); 2 Department of Natural Sciences, Novosibirsk State University, 2 Pirogova Street, 630090 Novosibirsk, Russia

**Keywords:** DNA repair, base excision repair, DNA glycosylases, noncatalytic protein domains, intrinsically disordered protein regions, protein–protein interactions, post-translational modifications, DNA binding, lesion search in DNA

## Abstract

Many proteins consist of two or more structural domains: separate parts that have a defined structure and function. For example, in enzymes, the catalytic activity is often localized in a core fragment, while other domains or disordered parts of the same protein participate in a number of regulatory processes. This situation is often observed in many DNA glycosylases, the proteins that remove damaged nucleobases thus initiating base excision DNA repair. This review covers the present knowledge about the functions and evolution of such noncatalytic parts in DNA glycosylases, mostly concerned with the human enzymes but also considering some unique members of this group coming from plants and prokaryotes.

## 1. Introduction

Many protein molecules in living cells consist of two or more structural domains, tens to hundreds of amino acids long [1,2]. A domain is usually defined as a separate protein part that has a defined structure and function and may evolve largely independently of the rest of the protein. In the case of enzymes, their catalytic activity is often localized in one or several domains, which form a core fragment, while other domains of the same protein may participate in activity regulation, protein–protein interactions, localization in the cell, etc.

In multidomain proteins, often, the function of the core fragment is known, whereas the roles of other domains are unclear. The development of methods for protein domain prediction based on homology and structural modeling [3,4,5] has led to the description of hundreds of domains of unknown functions. Moreover, there are many cases when a protein possesses known domains and long tails or linkers that are not identified as domains. Structurally, they may be true domains (i.e., have a defined structure and be separated from other domains), but more often, they are disordered and either not solved by X-ray crystallography or prevent crystallization and thus have to be removed to solve the core structure of the protein. This greatly complicates the analysis of their functions.

An important group of proteins that contain noncatalytic domains is DNA glycosylases, a class of enzymes participating in DNA base excision repair (BER) (Table 1). DNA glycosylases recognize their substrate-damaged bases and cleave the *N*-glycosidic bond between the base and C1′ of deoxyribose. How BER proceeds after that depends on the nature of the DNA glycosylase. Monofunctional DNA glycosylases stop after base excision, forming an apurinic/apyrimidinic (AP) site, which is further cleaved by AP endonucleases, introducing a break into the damaged strand 5′ to the AP site. Bifunctional DNA glycosylases possess an AP lyase activity, nicking DNA 3′ to the AP site by β-elimination, with AP endonucleases cleaving the modified deoxyribose off the 3′-end. Alternatively, some bifunctional DNA glycosylases catalyze β,δ-elimination, leave a 3′-terminal phosphate, and require polynucleotide kinase/3′-phosphatase (PNKP) for its removal. BER is completed by incorporation of an undamaged nucleotide and strand break ligation [6,7].

**Figure 1:**
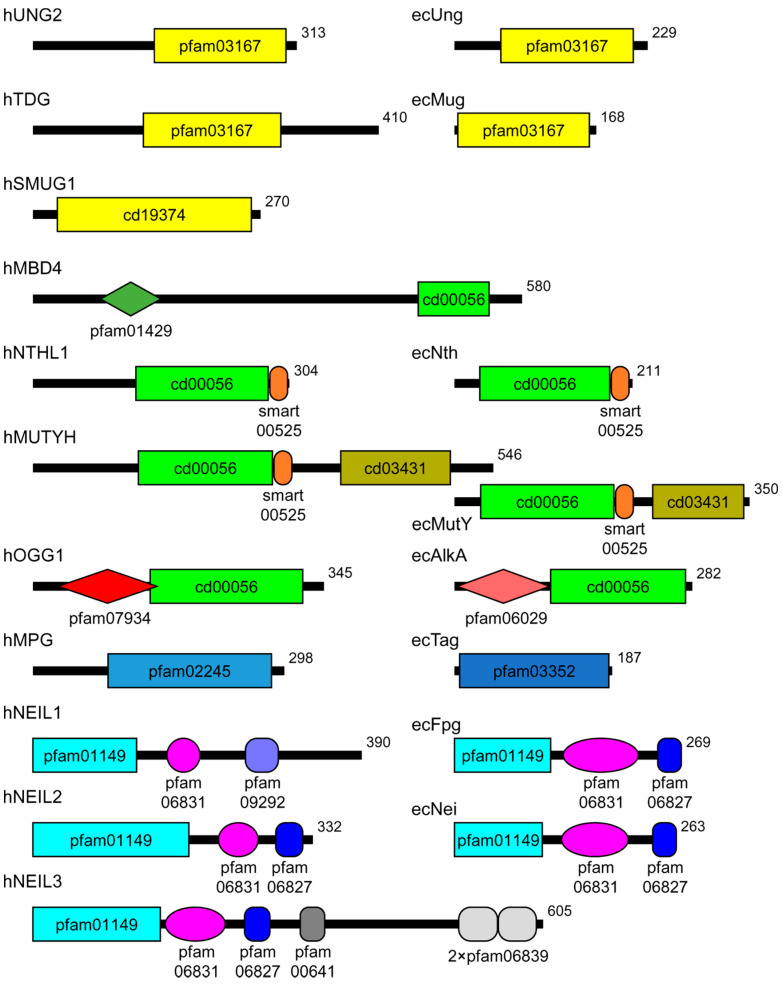
Scheme of the domain organization of human and *E. coli* DNA glycosylases. Human proteins are marked with the prefix “h”, *E. coli* proteins, with “ec”. The domains are demarcated according to the Conserved Domains Database [4]. The shown domains are: pfam03167 and cd19374, α/β-fold domain; pfam01429, methyl-binding domain; cd00056, HhH domain; smart00525, iron–sulfur cluster; cd03431, NUDIX domain; pfam07934, OGG1 N-terminal domain; pfam06029,AlkA N-terminal domain; pfam02245, FMT-C-like domain; pfam03352, methyladenine glycosylase domain; pfam01149, β-sandwich domain of H2TH proteins; pfam06381, H2TH domain; pfam09292, zincless finger; pfam06827, Fpg/IleRS zinc fingers; pfam00641, RanBP zinc finger; pfam06839, GRF zinc finger. In the human proteins, only one major isoform is shown.

Based on the structure of the core catalytic domain, most DNA glycosylases are divided into three superfamilies: α/β-fold (uracil-DNA glycosylase homologs), HhH (containing a helix–hairpin–helix DNA-binding motif), and H2TH (containing a helix–2 turns–helix DNA-binding motif) (Table 1, Figure 1) [8]. On the other hand, some glycosylases are sole members of narrower groups, such as human methylpurine–DNA glycosylase that belongs to the FMT_C family (homologs of the C-terminal domain of methionyl-tRNA formyltransferase). In certain cases, the catalytic function may be split between different domains; for example, in all H2TH DNA glycosylases, the active site residues belong to both the N-terminal β-sandwich domain, the H2TH domain, and the C-terminal zinc or zincless finger [9,10]. The active site of OGG1 is mostly formed by the HhH domain, while the critical interactions with the damaged base are mediated by the N-terminal domain [11].

Notably, many eukaryotic DNA glycosylases are different from their bacterial homologs in having additional sequences that have no role in catalysis (Figure 1); we henceforth call these sequences “noncatalytic domains” regardless of whether they are true structured domains. Oftentimes, these sequences can be removed without affecting the activity of the enzyme, and in many cases, their structure has not been solved. Nevertheless, the functions of these domains have been actively investigated by many biochemical approaches. In this review, we discuss the present state of knowledge regarding the noncatalytic domains of DNA glycosylases.

## 2. Unstructured Tails and Loops

**Figure 2:**
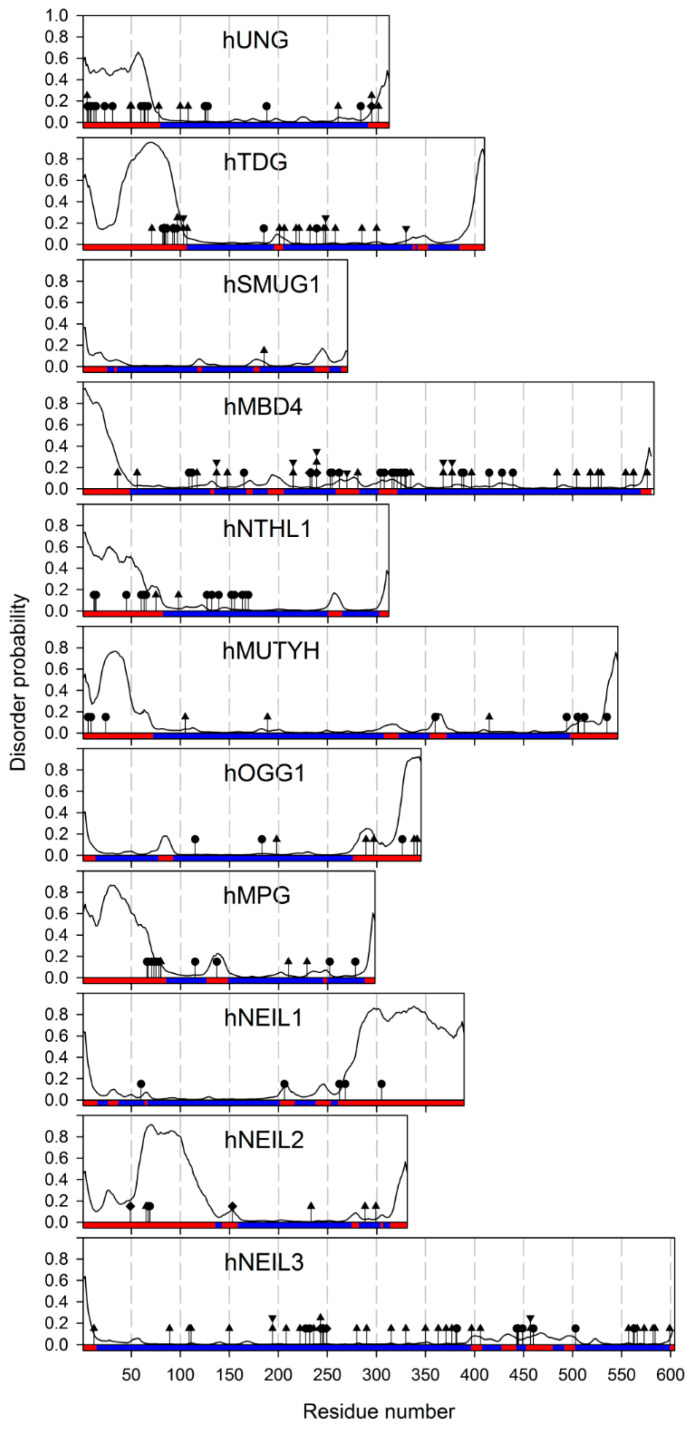
Predicted disorder and sites of post-translational modifications in human DNA glycosylases. The disorder probabilities were calculated using the ESpritz neural network [12]. The colored bar corresponds to ESpritz predictions: ordered (blue) and disordered (red). The sites of post-translational modifications are labeled by circles (Ser/Thr/Tyr phosphorylation), diamonds (Lys acetylation), triangles (Lys ubiquitylation), and reverse triangles (Lys sumoylation). The sites of modifications are taken from the PhosphoSitePlus proteomic database [13] and low-throughput studies discussed in the main text.

Many eukaryotic DNA glycosylases possess considerably long tails or internal fragments outside the established domains. Those are often referred to as “disordered” because of their generally low complexity (Figure 2). However, it is important to realize that the experimental evidence of the disorder exists only for a handful of these protein regions.

Uracil–DNA glycosylases, the first DNA glycosylases discovered in the 1970s, provide an essential example of the tails’ functions in DNA glycosylases. Uracil–DNA glycosylase removes uracil bases (Ura) from DNA. Ura, a canonical nucleobase in RNA, appears in DNA through deamination (spurious or targeted) of Cyt, or through incorporation of dUMP from the nucleotide pool, the latter source believed to be quantitatively more important [14]. Ura in genomic DNA can be mutagenic and disruptive for cell regulation, necessitating its quick repair [15,16]. Uracil–DNA glycosylases are termed Ung in *E. coli*, UNG in humans, and Udg in some other species; here, we will use Ung as a general name for the bacterial enzymes and UNG for eukaryotic ones. These enzymes can be found in all domains of life, including some viruses. Many of them are compact monomeric proteins, consisting essentially of a catalytic core. However, some UNG homologs acquired specific functions, as discussed below, and many eukaryotic UNGs possess likely disordered extensions entailed with regulatory and possibly mechanistic roles.

The human *UNG* gene produces two mRNA isoforms, *UNG1* and *UNG2*, which use alternative transcription start sites (reviewed in [17]) and are translated into polypeptides with different N-termini. *UNG1* codes for the UNG1 mitochondrial isoform (which also can be partly found in the nucleus), while the UNG2 protein isoform is exclusively nuclear. Accordingly, the unique N-terminal part of UNG1 carries a strong canonical amphiphilic helix mitochondrial targeting sequence [18,19]. The nuclear localization signal of UNG2 is less well defined, containing both a short basic residue-rich element from the unique N-terminus and some unspecified sequences from the noncatalytic part of the polypeptide common for UNG1 and UNG2 [18,19]. In addition, the N-terminal tail of UNG2 harbors protein–protein interaction sites with proliferating cells nuclear antigen (PCNA) and replication protein A (RPA) [20,21]. Moreover, the N-terminal tail of UNG2 is rich in the residues that undergo site-specific post-translational modifications. Many of these, such as Ser/Thr phosphorylation at Ser9, Ser12, Ser14, Thr31, Ser63, or Ser67, acetylation at Lys5, or ubiquitylation at Lys5, Lys49, Lys50, Lys78, Lys100, or Lys108 of UNG2 (Figure 2), are only observed in high-throughput functional proteomic experiments, and their exact function is unclear, although they overlap with protein–protein interaction sites [13]. Others have been studied in more detail. For example, phosphorylation of UNG2 at Thr6, Tyr8, and Thr126 following UV irradiation uncouples UNG from its complex with PCNA and RPA and promotes BER, while its dephosphorylation by PPM1D protein phosphatase suppresses BER [22,23]. Phosphorylation by cyclin-dependent kinases at Ser23, Thr60, and Ser64 is cell cycle regulated, with phospho-Ser23 promoting the recruitment of UNG2 to replication forks and increasing the enzyme turnover to allow quick U excision, and phospho-Thr60 and phospho-Ser64 targeting UNG2 for degradation upon entry to the G2 phase [24,25,26,27,28].

The N-terminal tail of human UNG2 appears to be truly unstructured in the isolated protein. Disorder in the residues 1–60 was established by NMR in the full-length protein, and the isolated 1–92 fragment appears to be almost completely disordered [29]. In a homologous protein, UNG from *Acanthamoeba polyphaga* mimivirus, a comparison of far-UV circular dichroism spectra of the full-length protein and a deletion mutant with residues 1–94 removed suggests that the N-terminal tail is predominantly random coil [30]. Interestingly, the tail of human UNG2 becomes more ordered under the conditions of macromolecular crowding, suggesting that the disorder may be restrained in the densely packed nuclear environment [31].

Two other families of the α/β-fold DNA glycosylase superfamily have been extensively studied. SMUG1 proteins, mostly limited to the Deuterostomia clade including vertebrates, are compact, constitutively expressed uracil–DNA glycosylases with the substrate specificity closely resembling UNG proteins but somewhat more permissive with respect to the nature of a substituting moiety at C5, e.g., capable of excising 5-hydroxymethyluracil in addition to U [32]. The catalytic domain constitutes most of the SMUG1 length (Figure 1). In contrast, the architecture of another family featuring eukaryotic TDG and bacterial Mug DNA glycosylases is similar to UNG/Ung; the bacterial protein does not have much beyond the catalytic domain, whereas TDG proteins possess long N- and C-terminal tails (Figure 1). As the primary role of TDG is in active epigenetic demethylation rather than genome protection from damage, these tails have multiple functions in the interactions with chromatin remodeling machinery and transcription factors (recently reviewed in [33]). Acetylation of TDG at several lysines in the N-terminal tail by CBP/p300 histone acetylase decreases the affinity of TDG for DNA, reduces its activity on T:G mispairs, and prevents its association with APEX1, the next enzyme in the BER pathway [34,35]. On the contrary, phosphorylation of Ser/Thr residues in the same region by protein kinase Cα does not affect the enzyme’s activity but is mutually exclusive with the acetylation [35]. NMR data also indicate a high degree of disorder in the N- and C-terminal tails of human TDG [36,37].

Another example of isoform-dependent tail function is presented by OGG1, a protein from the HhH structural superfamily. Alternative splicing of *OGG1* pre-mRNA produces two groups of isoforms, *OGG1-1* and *OGG1-2*, using exons 7 and 8, respectively, differing in their C-terminal tails ([38,39] and recently reviewed in [17]). The structure of the extensively studied nuclear protein isoform, OGG1-1a, shows that the isoform 1-specific sequence forms part of the lesion-binding site and ends in the last disordered 20 amino acid residues where a nuclear localization signal is located [11]. The N-terminal peptide that bears a mitochondrial import sequence common for all isoforms also appears to be disordered, as judged from its sensitivity to limited proteolysis [11]. In contrast, information on the major mitochondrial isoform OGG1-2a or any other group 2 isoforms is very limited, and no structure is available. OGG1-2a lacks the glycosylase activity when expressed in *E. coli* [40]. Yet, in human cells, it interacts with the inner mitochondrial membrane NADH:ubiquinone oxidoreductase 1 β subcomplex 10, a component of respiratory Complex I, and apparently participates in the mitochondrial 8-oxoguanine repair [41]. As the mitochondrial DNA is associated with the inner membrane [42], it is tempting to speculate that the C-terminal tail of OGG1-2a could form an alternative active site when bound to Complex I and participate in the repair right at the location where damaging reactive oxygen species leak from the respiratory chain. The functions of the mitochondrial OGG1 isoforms are certainly worth more attention.

In the H2TH structural superfamily, NEIL1 is predicted to have a significantly disordered C-terminal tail (Figure 2). Circular dichroism spectra of the isolated last 78 residues of human NEIL1, as well as small-angle X-ray scattering data from the full-length and C-terminally truncated protein confirm that this part is mostly disordered but is still able to fold back on the protein’s catalytic core, increasing the stability of the whole molecule [43,44,45]. Interestingly, the presence of osmolytes seems to induce folding of the C-terminal tail, which may suggest that NEIL1′s activity or localization could be regulated by liquid–liquid phase separation. This behavior resembles the crowding-induced ordering of the UNG2 N-terminal tail discussed above. As suggested by fluorescence spectra of full-length and truncated NEIL1, its tail might fold back onto the catalytic domain and stabilize its native conformation [43]. Additionally, the C-tail is involved in multiple NEIL1 interactions with downstream BER factors, poly(ADP-ribose) polymerase 1, and the nuclear and mitochondrial replication machinery [45,46,47,48,49,50,51,52,53], while the acetylation of the lysines in the tail by CBP/p300 is required for NEIL1 anchoring to nuclear chromatin [54]. Another H2TH protein, NEIL2, carries a long insert in its N-terminal domain, which is disordered in the X-ray structure of NEIL2 from gray short-tailed opossum *Monodelphis domestica* [55]. Hydrogen/deuterium exchange mass spectrometry experiments show very fast exchange of amido protons in this fragment, confirming its intrinsic disorder [56]. The role of this insert, unique among the H2TH family members, remains enigmatic.

The disordered tails are often regarded as modulators of glycosylases interaction with DNA. This role is supported by the measurements of glycosylase processivity, i.e., their ability to search for the damaged site by sliding along DNA without releasing it [57]. The N-terminal tail of human UNG2 was reported to enhance the enzyme’s processivity under physiological salt and crowding conditions [58,59,60] and to anchor the enzyme near ssDNA/dsDNA junctions, resembling replication forks and transcription bubbles [61]. The importance of the presumably disordered N-terminal tail for the processive lesion search was also shown for human MPG [62]. On larger distance scales, based on coarse-grained molecular modeling, disordered tails have been suggested to facilitate protein intersegment transfer between sites separated by a large distance along the DNA contour but brought together in 3D space [63,64]. However, when followed experimentally for MPG, intersegment transfer did not require the tail [65], so this mechanism remains only an attractive possibility thus far. Another role of the tails in the enzyme–DNA interactions may rely on selective tuning of the substrate or product binding affinity. The N-terminal tail of MPG seems to be involved in the enzyme turnover regulation; it strongly decreases the affinity of MPG for the AP site-containing DNA, allowing for faster product release [66]. Deletion of the N-terminal tail moderately decreased substrate cleavage by UNG from mimivirus [30]. An opposite effect was observed for the N-terminal tail of NTHL1: the truncated enzyme demonstrated much higher turnover [67,68]. Notably, the N-terminal tail of NTHL1 can directly interact with the AP site if the enzyme–product complex is stabilized on DNA by mutations uncoupling the glycosylase and AP lyase activities [69].

## 3. Zinc-Binding Structural Motifs

**Figure 3:**
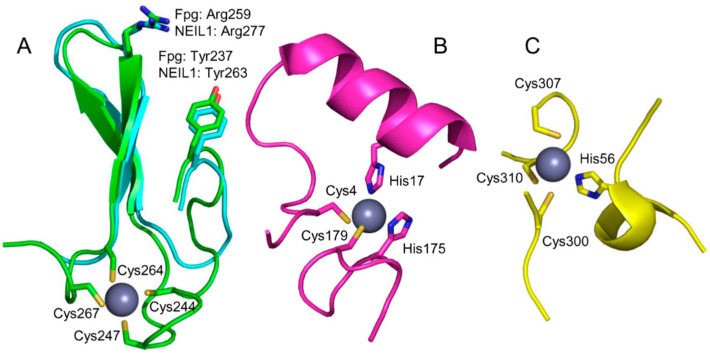
Zinc-binding motifs and their structural analogs in DNA glycosylases. (**A**) Superimposed zinc finger of *E. coli* Fpg (green; PDB ID 1K82 [72]) and zincless finger of human NEIL1 (cyan, PDB ID 5ITT [79]). (**B**) Zinc snap motif of *E. coli* Tag (PDB ID 1NKU [80]). (**C**) Zinc linchpin motif of mouse MUTYH (PDB ID 7EF8 [81]). The Zn^2+^ ion is shown as a gray ball. Zinc-binding residues are shown as sticks; the rest of the protein is omitted for clarity.

H2TH superfamily DNA glycosylases comprise two domains connected by a flexible linker; the active site is formed by residues from both domains [9]. The C-terminal half of the catalytic domain of many H2TH glycosylases (*E. coli* Fpg and Nei, human NEIL2 and NEIL3) is equipped with a prominent structural feature identified as a Cys_4_-type zinc finger in earlier works [70,71]. The X-ray structures [72,73] show that this part belongs to the β ribbon class of zinc fingers [74] (Figure 3A). NEIL2 proteins possess a Cys_3_His tetrad of Zn^2+^-coordinating residues, but otherwise, their zinc finger is very similar to those of Fpg, Nei, and NEIL3 [55,75]. However, later some members of the superfamily were identified (e.g., *Arabidopsis* MMH and human NEIL1) that bear an almost identical β-hairpin lacking Zn-coordinating residues [76,77], dubbed a “zincless finger” (Figure 3A). Interestingly, phosphoproteomic studies have identified phospho-Tyr263 in the zincless finger of NEIL1 (Figure 3A), and the corresponding phosphomimetic mutation Y263E completely inactivates the protein [78].

Unlike many conventional zinc fingers that recognize specific sequences in DNA and are often present as clustered units in the protein, the H2TH glycosylases’ fingers are solitary and contribute a single absolutely conserved Arg residue to the active site (Figure 3A) where it participates in a clamp pinching two phosphates that flank the damaged nucleoside. This pinching is a necessary step in the damaged nucleoside eversion mechanism required to flip the lesion out of the double helix and into the enzyme’s active site [73,75,82]. Except for this Arg, the analysis of intramolecular residue coevolution [83] and protein structure vibrational modes [84] in Fpg shows that the zinc finger is largely uncoupled from the rest of the protein, thus being a domain in the strict sense.

The closest sequence relatives of H2TH glycosylases’ zinc fingers are found in isoleucyl tRNA synthetases; however, they adopt a quite different structure with four short β-strands followed by an α-helix, do not interact with the nucleic acid [85], and probably should not be regarded as structural analogs.

NEIL3, the largest protein in the superfamily, possesses a long C-terminal extension that carries three additional β-ribbon zinc fingers different from the DNA-binding finger discussed above: a RanBP-type zinc finger and two GRF zinc fingers. GRF zinc fingers are present in many DNA-binding proteins, including another BER-related protein, APEX2, which hydrolyzes oxidatively damaged DNA in the 3′ → 5′ direction [86]. RanBP zinc fingers are mostly regarded as protein–protein-interacting units, as in the Ran-binding proteins in which they are involved in binding Ran/GDP [87]. The GRF zinc-finger-containing part of human NEIL3 has been crystallized, revealing a β-ribbon structure well suited for binding single-stranded DNA [88]. Interestingly, in the mouse protein, the GRF zinc fingers of NEIL3 efficiently bind single-stranded and forked DNA but inhibit the glycosylase activity, perhaps competing with the catalytic domain for substrate binding [88]. Forked DNA is a preferred substrate for NEIL3, possibly reflecting its role in the repair of stalled replication intermediates [89], and zinc-finger-mediated protein–protein and protein–DNA interactions within the replication fork might be critical for the correct positioning of NEIL3 to repair the lesions encountered during the replication.

Plant genomes code for several unusual DNA glycosylases, DEMETER (DML) and its homologs ROS1 (DML1), DML2, and DML3, which participate in active epigenetic demethylation through the direct incision of 5-methylcytosine (mC) from DNA [90,91]. The C-terminal part of these enzymes possesses a permuted CXXC-type zinc-finger homologous to zinc fingers present in several DNA methylation-related proteins such as MeCP2 mC-binding protein, MLL1 histone methyltransferase, and DNMT1 cytosine-5-methyltransferase [92]. The removal of the C-terminal part leads to ROS1 inactivation and loss of interactions with the H3 histone [93,94], but besides the zinc finger, this part of the protein contains an RNA recognition motif (discussed in Section 7) that may also participate in DNA binding. In the absence of the structure, the function of the zinc finger in DML-like DNA glycosylases remains unclear.

Two other DNA glycosylases were discovered to possess unusual zinc-binding sites, which was quite unexpected since neither one depends on Zn^2+^ for the enzyme activity. *E. coli* 3-methyladenine-DNA glycosylase I (Tag) was found to harbor a “zinc snap” motif [80]: two Cys and two His residues coming from the N- and C-terminal protein tails (Figure 3B). The Zn^2+^ ion is tightly coordinated and can be removed only after protein denaturation, suggesting that the zinc snap is a genuine metal-binding site. The coordinating residues are highly conserved, and the Zn^2+^ occupation is maintained in the structures of Tag homologs from *Salmonella enterica* [95] and *Staphylococcus aureus* [96]. Presumably, the zinc snap motif plays the structural role, helping to fold the protein chain, but too little experimental data is available to define its functions more confidently.

Another unanticipated Zn^2+^-binding DNA glycosylase is MUTYH, a eukaryotic homolog of the bacterial adenine–DNA glycosylase MutY. Both MUTYH and MutY contain three domains: a six-barrel domain and a FeS domain together constituting the catalytic core, and a C-terminal NUDIX-like domain that confers specificity for 8-oxoguanine opposite to the excised A [97,98]. However, the linker connecting the core and the NUDIX domain is much longer in MUTYH than in MutY. Three conserved Cys residues are located in the linker of human and mouse MUTYH, and the preparations of MUTYH contain substoichiometric amounts of Zn^2+^, which become negligible after the cysteines are replaced with serines [99,100]. This Zn^2+^ binding site was termed a “zinc linchpin” [99]. The nature of the fourth Zn^2+^ ligand is somewhat ambiguous: it was identified as Cys230 (human MUTYH numeration) from the quantum mechanics/molecular mechanics model coupled with site-directed mutagenesis data [100], while in the recently solved structure of mouse MUTYH, the fourth ligand is His56 (His71 in human MUTYH) [81] (Figure 3C). However, this region of the protein seems to be particularly structurally pliable, with even the two crystallization forms of mouse MUTYH showing different organization of the Zn^2+^ ligand shell (fully intramolecular vs. ligands coming from two protein molecules in the crystal cell) [81]. Mutations of the unambiguous Zn^2+^-coordinating Cys residues reduce the affinity of MUTYH for 8-oxoguanine-containing DNA and its ability to prevent mutagenesis when expressed in *E. coli* [99,100]. However, the truncated human MUTYH 65–350 lacks Zn^2+^ yet retains activity [101], and MutY homologs from bacteria and fission yeast lack the zinc linchpin motif altogether. The interdomain linker in MUTYH has been shown to mediate its interactions with APEX1, the next enzyme in the BER pathway, the 9-1-1 adapter complex, and SIRT6 protein deacetylase [101,102,103,104,105], although the role of Zn^2+^ in the protein partner binding has not been investigated. Thus, the zinc linchpin motif, while not required for the catalytic activity, might be important for specific tuning of MUTYH activity towards some substrates or for protein–protein interactions.

## 4. Iron–Sulfur Clusters

Many important proteins of cell metabolism, including nucleic acid metabolism, contain iron–sulfur clusters (FCL) [106,107]. These structural units have different stoichiometries ([2Fe–2S], [4Fe–3S], [3Fe–4S], and [4Fe–4S]), are usually electrochemically active, and often participate in redox reactions. Several DNA glycosylases possess [4Fe–4S] FCLs. The best studied of those are endonuclease III (Nth) and MutY, both belonging to the HhH structural superfamily [8]. *Micrococcus luteus* UV endonuclease and *Methanothermobacter thermautotrophicus* T:G DNA glycosylase (Mig.Mth) are two other examples of HhH DNA glycosylases containing an FCL; they are similar to Nth in their structure but have different substrate specificities [108,109]. The HhH superfamily also includes plant DME-like, which have a unique split Nth-like catalytic core with an FCL [91]. Besides the HhH superfamily, FCLs are present in two families of uracil–DNA glycosylases, namely Families 4 and 5, which mainly come from extremophilic species [110,111,112].

For a long time, FCL in DNA glycosylases were regarded as redox-inactive and having only a structural role, since the cluster damage by oxidation inactivates Nth, the prototypic FCL-containing glycosylase [113,114]. However, since the mid-2000s, a seminal series of studies by Barton and colleagues revealed that FCLs in Nth, MutY, *Archeoglobus fulgidus* Family 4 uracil–DNA glycosylase, and several repair proteins outside the BER pathway are not only redox-active, but their redox potential is similar to that observed in high-potential [4Fe–4S] ferredoxins, the bacterial proteins that participate in anaerobic electron transport [115,116,117,118,119,120,121,122,123,124]. The cluster cycles between the charge states [4Fe–4S]^2+^ (reduced, the ground state in the free protein) and [4Fe–4S]^3+^ (oxidized). However, the redox activity is only revealed upon DNA binding, which activates the cluster towards oxidation. Strikingly, in the oxidized state, FCL-containing proteins bind DNA 2–3 orders of magnitude more tightly than in the reduced state, presumably due to strengthened electrostatic interactions [125]. As a result of these studies, a hypothesis of DNA damage remote sensing emerged (summarized in [106]). In this model, an oxidative DNA lesion can oxidize the nearest randomly bound FCL-containing repair protein within a distance of up to a few hundred base pairs through π-stacking-mediated DNA charge transport. This oxidized protein molecule remains tightly bound to DNA and can in turn oxidize an FCL in another repair protein, and the process may be continued with the repair proteins gradually approaching the site of the damage. Notably, as the charge transport depends only on the presence of a redox-active FCL in the protein molecule, the oxidized nucleotide can thus attract not only BER enzymes, but also proteins from other DNA repair pathways, e.g., the nucleotide excision repair endonuclease UvrC [126] or the replication-coupled repair DNA helicase DinG [125]. Yet, many proteins participating in the removal of oxidized bases, such as *E. coli* Fpg and Nei and human OGG1 and NEILs, lack FCLs and are redox-inert, so the remote sensing model clearly cannot explain the full spectrum of oxidative damage repair.

One rather surprising exception from the behavior of FCL repair proteins is an Nth homolog from *Deinococcus radiodurans*. This bacterium, highly resistant to ionizing radiation and other kinds of abiotic stress, possesses three Nth homologs, of which two (Nth1 and Nth3) have been structurally characterized, whereas the third homolog, Nth2, shows the highest similarity to *E. coli* Nth of them all [127]. However, unlike in *E. coli* Nth and MutY, direct measurements of the FCL redox potential in *D. radiodurans* Nth2 revealed cycling between [4Fe–4S]^2+^ and [4Fe–4S]^+^ charge states, which was essentially independent of DNA binding [128,129]. The reasons for such discrepancy in the redox behavior of *E. coli* and *D. radiodurans* homologs presently remain unclear.

## 5. NUDIX Domain

**Figure 4:**
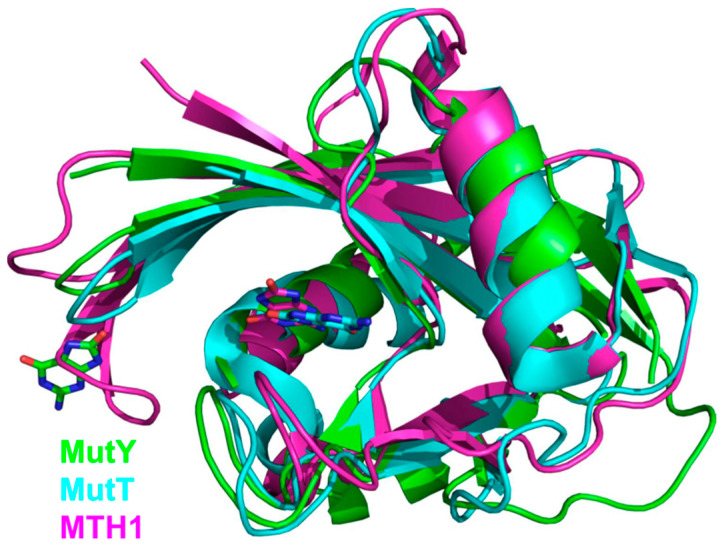
Overlay of NUDIX domains from *G. stearothermophilus* MutY (green; PDB ID 1RRQ [138]), *E. coli* MutT (cyan, PDB ID 3A6T [144]), and human MTH1 (magenta, PDB ID 3ZR0 [145]). OxoG bases at their respective binding sites are shown as stick models with carbon atoms colored the same as the respective protein.

MutY/MUTYH proteins provide a good example of a domain that had likely evolved as a functional protein on its own and was then grafted onto a pre-existing catalytic scaffold. These proteins are quite similar to Nth/NTHL1 but possess an additional domain that belongs to the NUDIX hydrolase (i.e., cleaving NUcleoside DIphosphates linked to X, where X is any moiety) superfamily. NUDIX enzymes hydrolyze a large variety of substrates of both nucleoside and non-nucleoside nature, such as NADH, CoA, diadenosine tetra- and hexaphosphates, ADP-ribose, metabolic nucleoside–sugars, mRNA caps, isopentenyl diphosphate, etc. [130,131]. Damaged dNTPs are an important group of substrates for NUDIX hydrolases [132]. *E. coli* MutT and human MTH1 (NUDT1) are members of the NUDIX superfamily that primarily hydrolyze 8-oxo-2′-deoxyguanosine (oxodGTP) to prevent its incorporation into DNA from the oxidized dNTP pool [133]. MutT and MTH1 participate in the so-called GO system, a subpathway within BER dedicated to cell protection against the mutagenic 8-oxoguanine (oxoG). This abundant oxidized purine presents its Hoogsteen face to DNA polymerases thus directing misincorporation of A during replication. The GO system, in addition to MutT/MTH1, involves an 8-oxoguanine–DNA glycosylase (Fpg, also known as MutM, in bacteria, OGG1 in eukaryotes) that removes oxoG from oxoG:C but not oxoG:A pairs, and adenine–DNA glycosylase MutY/MUTYH specific for A:oxoG and, to a lesser degree, A:G pairs [134,135]. The specificity of MutY/MUTYH enzymes for oxoG opposite the excised A base is provided by the C-terminal domain of the protein, which is not catalytic but is homologous to NUDIX enzymes, most closely to MutT proteins (Figure 4) [136,137]. Strikingly, despite this well-established role of the NUDIX domain in the MutY/MUTYH substrate specificity and the presence of a deep pocket suitable for nucleotide binding, the structures of *Geobacillus stearothermophilus* MutY (*Bst*MutY) and mouse MUTYH bound to their cognate DNA show that the mode of interactions of the glycosylase NUDIX domain and MutT/MTH1 with oxoG is quite different (Figure 4) [81,138,139,140]. The oxoG base remains fully intrahelical, assumes a *syn* conformation, and contacts the NUDIX domain only through N7 and O^8^ atoms making hydrogen bonds to a conserved Ser residue in a loop between two β-strands. However, the crystal structures may represent the later, low-energy recognition complex, and both stopped-flow kinetic experiments with a fluorescent reporter and chemical- or photo-crosslinking suggest that oxoG could be extrahelical at earlier stages of its recognition by MutY [141,142]. The structural nature of such an intermediate, if it actually exists, remains to be established. In a complex with undamaged DNA, the NUDIX domain of MutY assumes multiple conformations and cannot be clearly resolved by X-ray crystallography, although small-angle X-ray scattering data suggest that it still contacts DNA [143].

The NUDIX domain is dispensable for MutY/MUTYH catalytic activity, but its removal impairs the substrate properties of A:oxoG pairs that become similar to A:G in terms of the processing efficiency [146,147]. Structurally, the elimination of oxoG contacts with the NUDIX domain causes oxoG to be in an *anti* conformation just like the undamaged G, consistent with the kinetic effect [143].

Human MUTYH is a known tumor suppressor, and homozygous or compound heterozygous inactivating mutations in the *MUTYH* gene greatly increase the risk of colorectal cancer [97,148]. One of the mutations commonly found in human tumors is Gly382Asp located in the NUDIX domain. Biochemically, the mutant protein has lower activity than the wild-type one, although it is not completely inactivated; in fact, the cleavage of A:G substrates is affected to a greater degree than of A:oxoG [148,149,150]. In the *Bst*MutY/DNA and mouse MUTYH/DNA structures, the main chain amide of the homologous Gly residue coordinates the phosphate of the nucleotide located 5′ next to oxoG [81,138], so substitution of the negatively charged Asp for Gly likely disrupts this apparently important interaction. Thus, the NUDIX domain does not only participate in the recognition of oxoG but helps to mold the DNA into a bent shape observed in the pre-catalytic complex.

Phosphorylation of Ser524 in the NUDIX domain has been detected *in cellulo*, but its significance is unclear since both phosphomimetic and phosphoablating mutants have similar enzymatic properties [151].

## 6. Methyl-Binding Domains

Methyl-CpG-binding domain protein 4 (MBD4) is a DNA glycosylase consisting of an HhH superfamily catalytic domain and a methyl-CpG-binding domain (MBD) [152,153]. MBD is a small domain not found in other glycosylases but present in several DNA-binding proteins (MeCP2, MBD1, MBD3, MBD4) that regulate chromatin condensation and transcription status, often as parts of large multiprotein complexes involving histone deacetylases [154,155,156]. In fact, MBD4 also represses transcription from hypermethylated promoters in a histone-deacetylase-dependent manner, apparently independently of its DNA repair function [157,158]. Additionally, binding of MBD4 to mC-rich heterochromatin recruits a E3 ubiquitin ligase UHRF1 and a deubiquitylase USP7, both of which regulate the stability of DNMT1, the maintenance C5-methyltransferase [159].

Possible transcription regulation notwithstanding, MBD in MBD4 is mostly regarded as a domain that targets its DNA repair function to methylated CpG sequences. Full-length MBD4 preferentially excises T and U from mispairs with G in the 5′-(T/U)G-3′/3′-GC-5′ and 5′-(T/U)G-3′/3′-GmC-5′ contexts. The removal of MBD does not affect the enzyme’s activity [160,161]. A natural splice isoform skipping MBD and most of the interdomain linker was reported to retain its uracil glycosylase activity but lose the ability to excise mismatched T [162]. Interestingly, plant homologs of MBD4 lack an MBD but retain the long N-terminal extension [163]. Alternative splicing in this region produces protein isoforms with different intranuclear localization and different redistribution response to heat stress [164].

**Figure 5:**
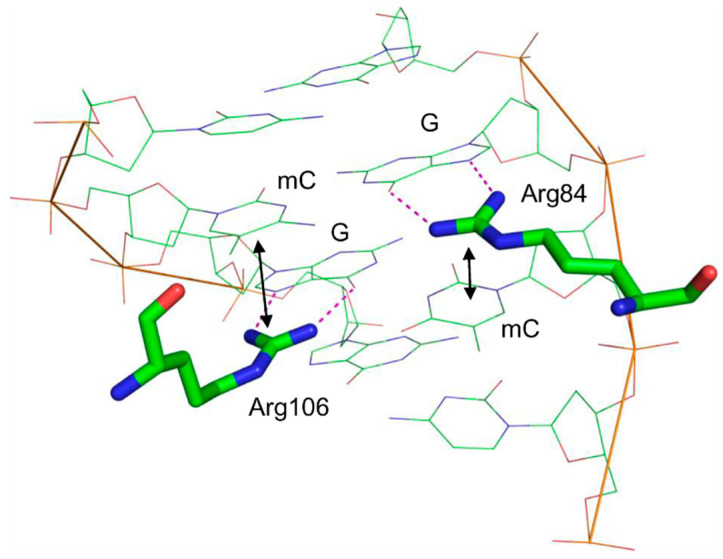
Structure of the mouse MBD4 methyl-binding domain bound to a fully methylated CpG site in DNA (PDB ID 3VXV [165]). Dotted lines indicate the hydrogen bonds formed between G and the critical Arg residues; double-headed arrows show stacking between these arginines and mC.

NMR and X-ray data on the structure of human and mouse MBD provide rationalization for the mechanism of methylated DNA recognition [165,166]. The key interactions are made through two Arg residues that both donate Nη1/2[Arg]…N7[Gua] and Nη1/2[Arg]…O^6^[Gua] hydrogen bonds to both guanines in the CpG dinucleotide (Figure 5). This interaction presses the guanine bases deeper towards the minor groove, allowing the π system of the arginines’ guanidine groups to stack with the adjacent pyrimidines. Apparently, the larger area of stacking provides binding preference for mC compared with C and allows the protein to recognize CpGs containing other modified pyrimidines such as 5-hydroxycytosine or T.

How MBD-driven localization of MBD4 to mCpG-rich regions is mechanistically coupled with the DNA repair function is still an open question. Inhibition of full-length MDB4 by isolated MBD on substrates containing a single methylated target site has been reported [167], suggesting that MBD may compete with the catalytic domain for the damaged CpG site but may be diverted by the presence of an undamaged methylated CpG site nearby. However, in a naked 60-bp fragment bearing seven fully methylated CpG dinucleotides and a central T:G mismatch, no activity enhancement was observed compared with nonmethylated DNA [168].

Most protein–protein interactions of MBD4 are mediated by the interdomain region, which, unlike the disordered tails of many DNA glycosylases, is predicted to be mostly structured (Figure 2). MBD4 forms a complex with the DNA mismatch repair protein MLH1 and Fas-associated death domain protein (FADD), and these interactions are apparently required to promote mismatch repair-directed apoptosis initiated by certain types of DNA damage, e.g., extensive 5-fluorouracil incorporation [153,169,170]. 5-Fluorouracil as well as *N*-methyl-*N*-nitrosourea and cisplatin induce sumoylation of MBD4 at lysines 137, 215, and 377 in the interdomain linker, which stimulates the enzyme’s activity [171]. Phosphorylation of the Ser165 and Ser262 in the interdomain linker by protein kinase C is also stimulatory [172].

## 7. RNA-Binding Elements

**Figure 6:**
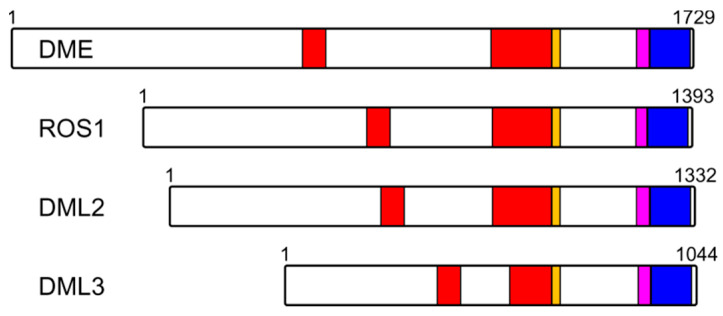
Organization of plant DME-like proteins. Red, split Nth-like domain (HhH superfamily); Orange, FeS cluster; Magenta, permuted CXXC zinc finger; Blue, RRM_DME motif. Numbering corresponds to the *A. thaliana* species.

The intersection between the cellular RNA milieu and BER is an area of acute interest, although many more questions than answers remain at present [173,174,175]. The only group of DNA glycosylases in which RNA-binding domains are identified is composed of DME-like plant epigenetic 5-methylcytosine–DNA glycosylases (Figure 6, see also Section 3) [92,176,177]. They possess a C-terminal domain designated as an RNA recognition motif (RRM_DME), although the homologous structural elements in different proteins recognize not only RNA, but also single-stranded DNA [178]. No data on its function or structure are available, except for studies of ROS1 with the deleted C-terminal part spanning both RRM_DME and the permuted CXXC zinc-finger domains; such a truncation, as mentioned above, inactivates ROS1 and interferes with nucleosome binding [93,94]. However, as the establishment of DNA methylation at many loci in the plant genome is targeted by small RNAs [90] and ROS1 preferentially demethylates these sites rather than those methylated in an RNA-independent manner [179], it is tempting to speculate that RRM-DME might somehow mediate active demethylation targeting through interactions with small RNAs.

In animals, the role of RNA in BER-dependent active demethylation is even less clear. Unlike in plants, demethylation in vertebrates depends on mC oxidation by TET family dioxygenases followed by processing via the BER pathway, in which TDG is the main initiating glycosylase [180]. Early reports on a TDG-like activity in chicken cells claimed that it also can directly remove mC [181,182], but this is now believed to be due to a co-purifying demethylation complex [183,184,185]. Notably, the active demethylation complex purified from the cells was reported to contain an unidentified RNA that targeted demethylation, together with an RNA helicase [186,187,188,189]. Later, the presence of RNA in a TDG–DNMT3b complex was confirmed in human cells [190]. Finally, the recent identification of TETILA, a long noncoding RNA directly interacting with TDG in a human TET2–TDG complex [191], resurrected the idea that active demethylation in animals might indeed recruit RNA as one of the active components, even if not for complementarity-based targeting. It is still unknown, however, what part of TDG binds RNA; given that the N-terminal tail is required to efficiently cleave T:G but not U:G substrates by TDG [192] and that the associated RNA stimulates T:G cleavage [190], the interaction could involve the N-tail.

## 8. Conclusions

DNA glycosylases present a multitude of functions associated with the noncatalytic domains, both structured and not. In some cases, such as the methyl-binding domain of MBD4 or FeS clusters, these functions are both expected from the role of the glycosylase and conserved structurally in proteins outside of DNA repair. In other cases (zinc fingers, NUDIX domains), the function may be expected, but its structural implementation is unique for DNA glycosylases, suggesting that an ancient fold was adopted and evolved to play a role different from its original purpose. Intriguingly, some noncatalytic domains of DNA glycosylases are clearly related to folds of a known function, but that function has not been confirmed for the glycosylases. For example, OGG1 and AlkA have an N-terminal domain that resembles the structure of TATA-box binding protein (TBP), but the reason for this similarity is totally obscure. Finally, a group of unstructured tails appears to be important for DNA glycosylase localization, cell cycle regulation, protein–protein interactions, and DNA binding, but their disordered nature complicates the analysis of their functions. These elements seem to be responsive to macromolecular crowding, suggesting that they might be involved in the regulation of subcompartment localization through liquid phase separation, a process that has drawn much attention recently. Overall, noncatalytic domains of DNA glycosylases represent a rich source of functionalities that can be targeted by drugs or serve as parts for protein engineering.

## Figures and Tables

**Table 1:** DNA glycosylases from humans and *E. coli*.

Enzyme	Substrate Specificity	Structural Superfamily
*Human*		
UNG	U in single- and double-stranded DNA, any context	α/β-fold
TDG	T, U, 3,*N*^4^-ethenoC and oxidized/deaminated derivatives of 5-methylC opposite to G in XpG dinucleotides	α/β-fold
SMUG1	U in single- and double-stranded DNA, any context	α/β-fold
MBD4	T and U opposite to G in XpG dinucleotides	HhH
NTHL1	Oxidized pyrimidines	HhH
MUTYH	A and 2-OH-A opposite to G or 8-oxoguanine	HhH
OGG1	8-oxoguanine and FapyG opposite to C	HhH
MPG	Ring-alkylated purines, hypoxanthine, 1,*N*^6^-ethenoA	FMT_C
NEIL1	Oxidized pyrimidines and purines, ring-open *N*7-alkylated G modifications, psoralen cross-links	H2TH
NEIL2	Oxidized pyrimidines and purines in bubble DNA	H2TH
NEIL3	Oxidized pyrimidines and purines in single-stranded DNA	H2TH
*E. coli*		
Ung	U in single- and double-stranded DNA, any context	α/β-fold
Mug	U and 3,*N*^4^-ethenoC opposite to G	α/β-fold
Nth	Oxidized pyrimidines	HhH
MutY	A opposite to G or 8-oxoguanine	HhH
AlkA	Ring-alkylated purines, hypoxanthine, 1,*N*^6^-ethenoA	HhH
Tag	Ring-alkylated purines	HhH ^1^
Fpg	8-oxoguanine opposite to C	H2TH
Nei	Oxidized pyrimidines	H2TH

^1^ Tag is distally related to the HhH group, lacking several key structural elements present in other superfamily members.

## Data Availability

The paper does not report any original data.
